# Fabrication and Characterization of Gel Beads of Whey Isolate Protein–Pectin Complex for Loading Quercetin and Their Digestion Release

**DOI:** 10.3390/gels8010018

**Published:** 2021-12-26

**Authors:** Xu Wang, Huaping Xie, Chenshan Shi, Piotr Dziugan, Hongfei Zhao, Bolin Zhang

**Affiliations:** 1 Beijing Key Laboratory of Forestry Food Processing and Safety, Department of Food Science, College of Biological Sciences and Biotechnology, Beijing Forestry University, Beijing 100083, China; kwonlee0818@163.com (X.W.); cxtc2000@163.com (H.X.); shichenshan@163.com (C.S.); zhaohf518@163.com (H.Z.); 2 Institute of Fermentation Technology & Microbiology, Poland Technical University of Lodz, 90924 Lodz, Poland; piotr.dziugan@p.lodz.pl

**Keywords:** emulsion gel beads, quercetin, structure, digestion, release

## Abstract

In this study, emulsion gel beads for loading quercetin were prepared through an emulsification/gelation process using whey protein isolate (WPI) and pectin. Emulsion gel beads’ properties were investigated by different pectin content. Additionally, the physicochemical properties, morphology and quercetin release properties from beads were explored. Firstly, electrical characteristics and the rheology of bead-forming solutions were measured, revealing that all systems had strong negative charge and exhibited shear-thinning behavior. The textural results demonstrated that the properties of emulsion gel beads were improved with increasing the content of pectin. It was also confirmed that crosslinking was formed between WPI emulsion and pectin by Fourier Transform Infrared (FTIR) analysis and thermogravimetric analysis (TGA). In addition, the shape of the beads was spherical or ellipses with smooth surfaces and they had a tight gel network of internal structures, which was visualized by using electron microscopy (SEM). Finally, the amount of quercetin released in vitro was gradually decreased with increasing pectin content; it was as low as 0.59%. These results revealed that WPI emulsion–pectin gel beads might be an effective delivery system for quercetin as a colon target and are worth exploring further.

## 1. Introduction

Quercetin (3,3′,4′,5,7-pentahydroxyflavone), a plant flavonoid, exists in a variety of vegetables, fruit and grains. Recently, quercetin has attracted considerable attention due to its beneficial biological activities, such as antioxidative, anti-inflammatory, antibacterial and anticancer effects [1,2]. However, the application of quercetin in the food industry and human gastrointestinal tract is limited due to its poor water solubility, chemical instability and low bioaccessibility [3]. Therefore, it is important to improve the chemical stability and bioaccessibility of quercetin and ensure the goal of slow release.

Recently, several delivery systems have exhibited that the bioaccessibility of quercetin can be improved, including emulsions and hydrogels, etc. [4,5,6]. Emulsions are a type of colloidal dispersion, which are usually prepared by protein and/or polysaccharides and form an amphipathic spherical core-shell structure so that fat-soluble nutrient substances such as quercetin can be encapsulated into the hydrophobic core [7,8]. Some globular proteins such as whey isolate protein (WPI) have good emulsifying properties [9,10]. Stable quercetin emulsions were prepared by using natural milk protein surfactants, which showed that the solubility and bioaccessibility of quercetin were greatly enhanced [5]. Quercetin and gallic acid were incorporated in the internal and the external aqueous phase of double emulsions, respectively, improving the physical stability and antioxidant activity of emulsions [6].

Hydrogels can absorb a great deal of water due to their three-dimensional network structure, making them ideal materials for functional food or pharmaceutical applications when they are swelled under physiological environments [11]. Among them, hydrogel beads have become functionalized owing to their core-shell structure, which could protect bioactivity against a harsh pH and gastrointestinal (GI) enzymes through the oral route, maintaining the required release time and increasing bioactive efficiency and absorption, so that it could be absorbed and employed by the human body to the utmost extent [12,13]. Pectin has been used to prepare hydrogel beads for delivery systems, showing good biocompatibility and biodegradability. Traditionally, pectin is dropped into the divalent cation solution to induce crosslinking of polymer chains through external gelation [7]. However, the diameter of pectin beads based on that approach is large if there are no techniques such as jet-cutter, membrane or pressure-solvent processes. Additionally, the diameter of the beads is an important factor in the stability and encapsulation efficiency [14]. It has been reported that an effective way of decreasing the size of beads is microencapsulation by emulsion, which comprises the combination of an encapsulating agent such as protein and oil phase with or without active ingredient, where the crosslinking formed via internal gelation [7,15]. Loyeau et al. reported encapsulated canola oil and potential probiotic bacteria through an emulsification/gelation process using whey proteins isolate (WPI)/dextran (DX) conjugate obtained by the Maillard reaction as emulsifiers, improving the stability and encapsulation efficiency of canola oil in WPI/DX conjugate emulsion gel beads [4]. To the best of our knowledge, few have studied the properties of emulsion gel beads containing quercetin; these performances may be critical for the development of quercetin delivery systems. Hence, it is important to choose a proper matrix material and emulsifier in structuring emulsion gel beads.

In fact, proteins (e.g., whey protein isolate (WPI)) and polysaccharides (e.g., pectin) have been widely investigated as gelling agents. WPI contains more positively charged amino acids (267.9 mg/g) at pH 7.0 (Tang, Ten, Wang, and Yang, 2006)), which are easy to react with anionic polysaccharides through electrostatic attractions. Moreover, WPI has higher solubility and surface hydrophobicity. Several reports have shown that protein solubility affects the interactions between protein and water, and surface hydrophobicity has an impact on protein–oil interactions on droplet surfaces (Loyeau et al., 2021; Wang et al., 2018; Tang et al., 2006). Simultaneously, pectin can improve the stability of emulsion through slowing down the movement of droplets, decreasing the collision frequency and coalescence rate, which are attributed to increasing the viscosity or forming a gel network in continuous phase. Therefore, the purpose of this study was to investigate the properties of emulsion gel beads loaded with quercetin. WPI and pectin were used as emulsifiers and structural strengthening agents, respectively. The encapsulation of quercetin in WPI emulsion–pectin gel beads was characterized and the release behavior of quercetin in the simulated gastrointestinal tract was estimated. The developed emulsion gel beads will have broad application prospects for delivering and controlling the release of active substances in the food and pharmaceutical industries.

## 2. Results and Discussion

### 2.1. Electrical Characteristics and Rheology of Bead Forming Solutions

**Figure 1:**
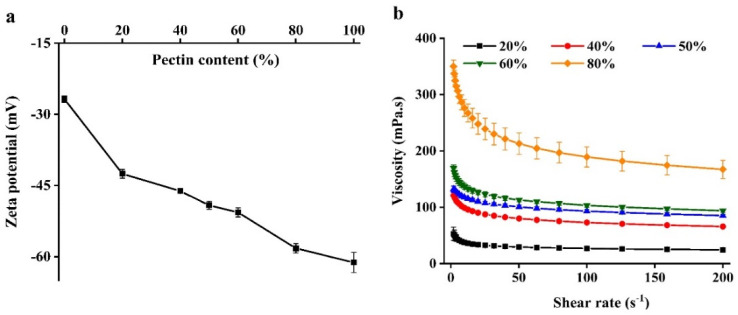
The zeta-potential (**a**) and apparent viscosity (**b**) of beads formation solutions used to prepare the hydrogel beads.

The electrical characteristics of WPI emulsion–pectin complex were tested because these determine the nature of the electrostatic interactions between biopolymers [16]. All beads with different pectin content had a highly negative charge; the solutions had a more negative surface potential when pectin content increased (Figure 1a). This effect can be attributed to the carboxylic acid group in the galacturonic acid of pectin dissociated to -COO^−^ when dissolved in water [17].

In order to assess the rheological properties of the WPI emulsion–pectin complex solutions whether impacted the formation of beads or not, their apparent viscosity were measured (Figure 1b). There was a previous study reported that the size of beads formed by atomization increased as the viscosity of complex solutions increased [18]. Additionally, some studies had also reported that shear thinning behavior of complexes exhibited a stronger resistance to elongational at high deformations [18,19]. For example, a report had studied that the effect of solution viscosity on bead formation, showing that the complex of alginate solutions and whey protein solution increased the shear thinning behavior, which caused the production of larger beads after injection [20]. It could be seen that all complexes showed shear-thinning behavior in the Figure 1b. Nevertheless, there were distinct differences in the apparent viscosity depending on the content of pectin, indicating that the viscosity did not have a crucial impact on bead formation. Containing 20% pectin of beads exhibited a lower degree of shear-thinning behavior than others owing to WPI emulsion was dominated in this system, fluid extension and breakup occurs more easily for more Newtonian liquids during shear flow [9].

### 2.2. Physical Properties of Beads

**Figure 2:**
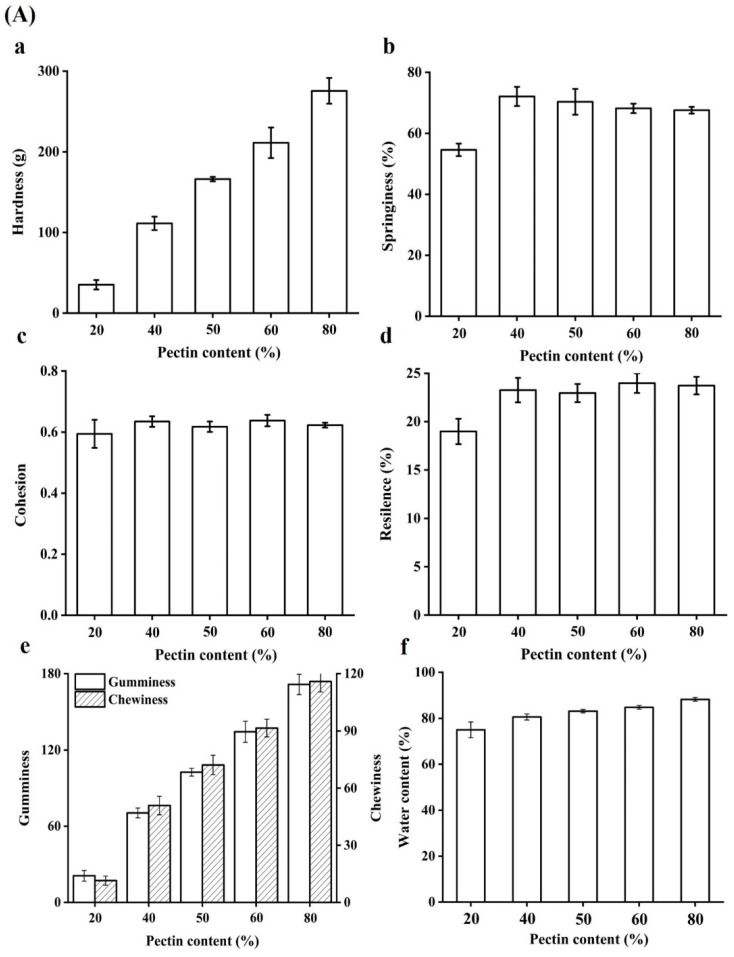

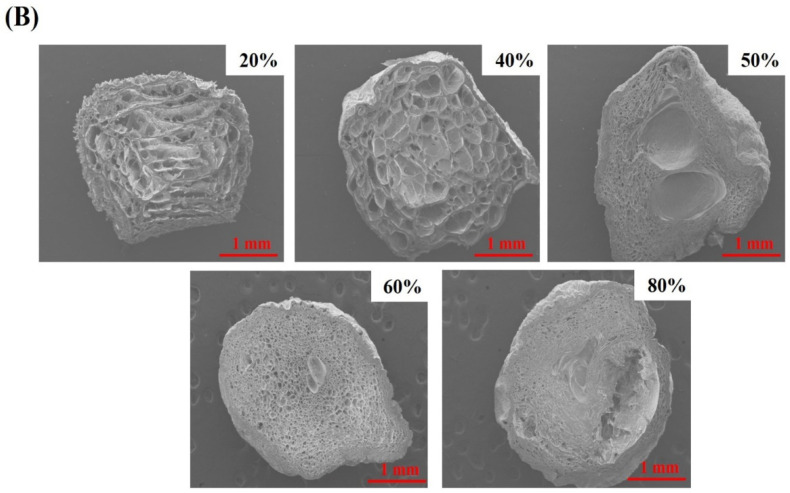
(**A**) Textural properties of gel beads: The hardness (a), springiness (b), cohesion (c), resilence (d), gumminess and chewiness (e) of quercetin-loaded beads with different content of pectin; Effect of pectin content on the water content in hydrogel beads (f). (**B**) SEM image (30×) of cross section of gel beads with different pectin content.

The texture characteristics of beads were measured. Hardness is regarded as the force required to attain a certain deformation, and it serves as a measure of food firmness [21]. As shown in Figure 2A, the hardness values (Figure 2a) were increasing with the content of pectin in beads increasing resulted with firmer structure. The reason may be the interstices of beads was smaller and smaller (Figure 2B). Moreover, 80% pectin exhibited the highest hardness value, suggesting the network between WPI and pectin was weak and it tend to become solid sphere from porous sphere (Figure 2B). Indeed, the springiness of all beads was increased, suggesting that adding pectin could improved the springiness of beads (Figure 2b). It had been reported that hydrogel beads should be had higher hardness and springiness so that these encapsulates containing active compounds could be applied in functional food products [22]. Cohesion is a force to the tested food can be deformed when it ruptures, and it is often used as the strength of internal bonds [21]. It showed that the cohesion values in all beads were no significant difference (Figure 2c). In addition, it could be seen that the resilience of beads was increased as pectin content increasing (Figure 2d). Chewiness is a product of hardness, cohesiveness and springiness, which is an energy needed to masticate solid food to a state ready for swallowing, reflecting the gel’s mechanical properties and provide references for evaluating subjective senses while masticating the gel [23]. Gel beads with different pectin content differed significantly in their texture property, and both the gumminess and chewiness of the high content of pectin beads were significantly higher than that of the low content (Figure 2e). The reason was that pectin molecules are highly branched with a vast number of branches and relatively large molecular weights [24]. The molecular structure of pectin usually gives food with a different texture. The increase in chewiness of beads upon pectin addition could be due to the viscosity of pectin which could be further strengthened the structure of beads. As for water content of beads (Figure 2f), all pectin-based beads contained more than 70% water and it could be up to 88.73% in beads with 80% pectin, indicating that the structure of 80% pectin beads might be less porosity, which would be slowed the permeability of active components from the particles [22].

### 2.3. FTIR Analysis

**Figure 3:**
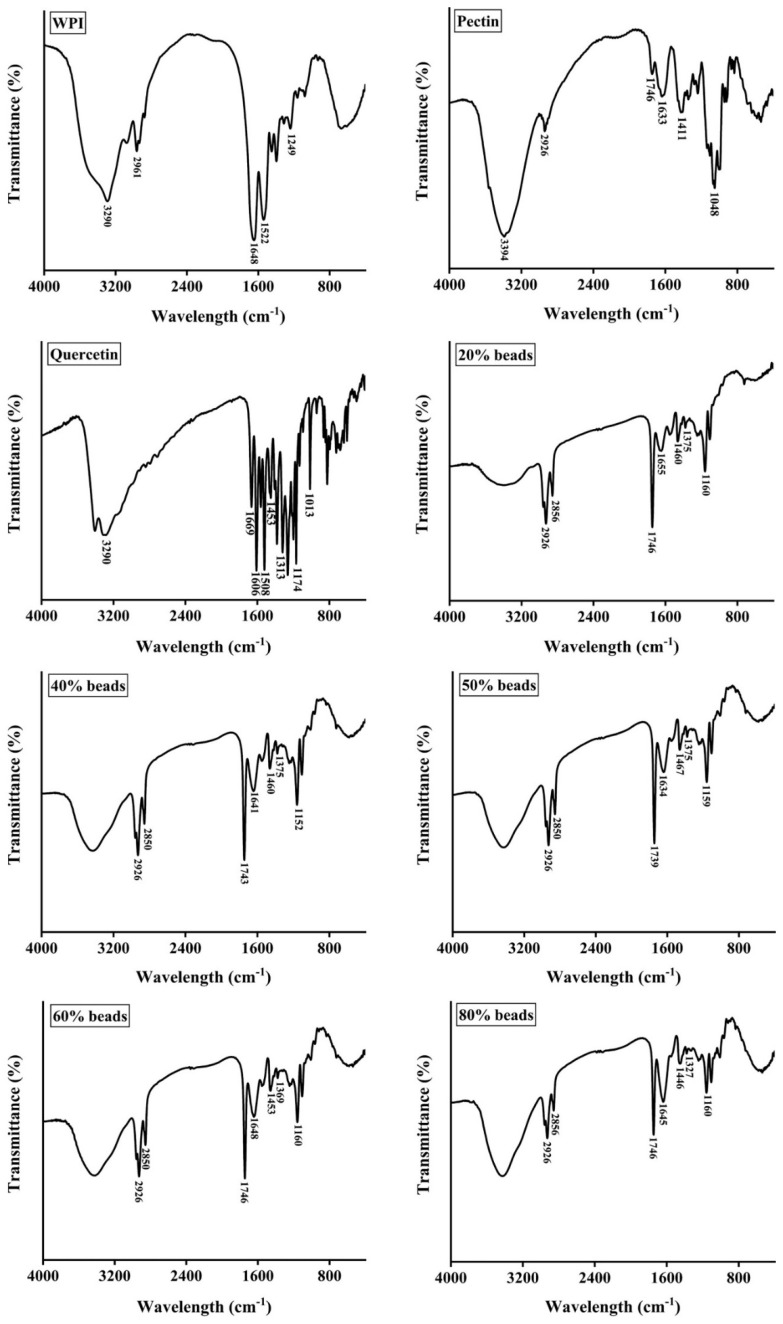
FTIR spectra of whey protein isolate (WPI), pectin, quercetin, and hydrogel beads. 20% beads, 40% beads, 50% beads, 60% beads and 80% beads represent hydrogel beads produced by WPI emulsion and different content of pectin (20%, 40%, 50%, 60% and 80%, respectively).

As shown in Figure 3, the FTIR spectra of WPI, pectin, quercetin, 20% beads, 40% beads, 50% beads, 60% beads and 80% beads. For WPI, the characteristic peaks of FTIR spectrum were observed at 1648, 1522, and 1249 cm^−1^, which represented the carbonyl (C=O) stretching of the secondary amide (amide I band), the bending vibrations of the N-H (amide II band) and the N–H stretching (amide III band), respectively. The absorption bands of pectin was detected at 3394, 2926, 1746, 1411 and 1048 cm^−1^ corresponding to the stretching vibrations of hydroxyl groups, the stretching vibrations of –CH bonds of methyl esters groups or pyranoid ring carbons, the carbonyl (C=O) stretching of the methyl esterified carboxyl groups, asymmetric stretching vibrations of –C–O–C– bonds and −CH groups or methyl esters groups and skeletal C–O and C–C vibration bands of glycosidic bonds and a pyranoid ring, respectively [25,26]. As for quercetin, the spectral characteristic peaks were shown at 3290, 1669, 1606, 1508, 1453, 1174, and 101 cm^−1^, which were stood for –OH stretching, aromatic ketonic carbonyl stretching, aromatic bending, C=C stretching of the aromatic ring, –OH phenolic bending, and aromatic stretching, respectively [27,28].

Furthermore, it was clearly shown that a new peak of 20%, 40%, 50%, 60% and 80% pectin beads was appeared around at 2850 cm^−1^, suggesting that the cross-linking existed between WPI and pectin. In addition, the peaks at 1633 and 1411 cm^−1^ of pectin were shifted to around 1650 and 1460 cm^−1^, respectively, indicating that carboxyl groups (–COO^−^) were cross-linked with Ca^2+^ [29]. The encapsulation of quercetin was identified by the appearance of the peak at around 1375 cm^−1^ which was a characteristic peak of quercetin (–OH phenolic bending, 1313 cm^−1^). However, it was interested that the peak of 80% pectin beads shifted to 1327 cm^−1^ because the fraction of WPI emulsion decreased.

### 2.4. TGA and Swelling Behavior of Beads

**Figure 4:**
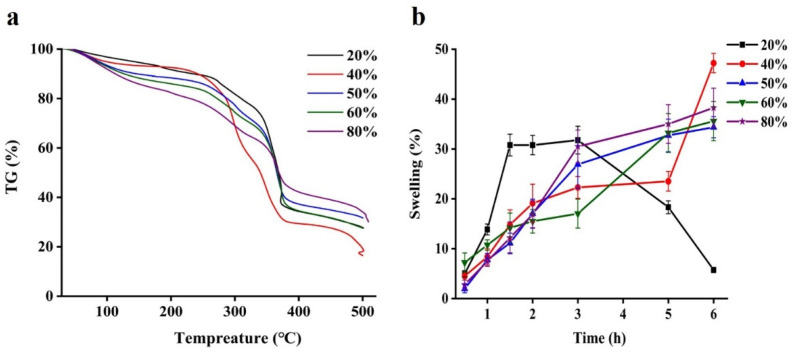
Thermogravimetric analysis curves (**a**) and water content (**b**) of quercetin-loaded beads with different content of pectin.

A thermogravimetric analysis of all beads as shown in the Figure 4a. The weight loss of all samples at around 100 °C was owing to evaporation of moisture. Beads displayed two weight loss. The first is in the range 100–260 °C could be attributed to the moisture loss from the WPI-pectin complex structure [26,30], and another thermal degradation between 260 and 370 °C might be quercetin [31]. It was noticeable that the mass loss of 40% pectin beads was the biggest in all beads, which could be explained considering that more residual water was entrapped in network structure formed by WPI and pectin. The last mass loss stage started about 370 °C might be needed a higher calcination temperature to remove all the organic matter.

The swelling degree is a crucial factor in the release of drugs from delivery systems [32]. Gel beads with a small range of swelling ratios serves as moisture immersion barrier, reducing quercetin release from the gel beads to surrounding media [13]. In the Figure 4b, the swelling properties of all beads in distill water after soaking at 25 °C for 6 h were measured. It could be easily to find that the swelling ratio of majority of beads gradually increased as the soaking time was prolonged, except for 20% pectin beads. Among them, beads containing 40% pectin show the highest swelling ratio after 6 h up to 48.5%, indicating that its structure was porous. However, the swelling ratio of beads with 20% pectin was distinctly decreased after 3 h, it was related to absorbed water molecules, which made network become expand and finally the polymer will be diluted and lose its structural integrity [33,34]. Therefore, it would be an effective means to regulate the bioactive release behavior through controlling the swelling of the hydrogel beads [34].

### 2.5. Morphology Study

**Figure 5:**
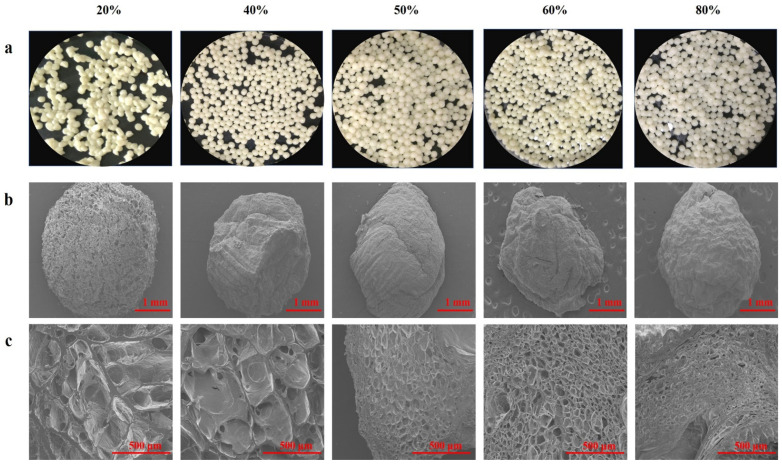
Images of quercetin-loaded beads with different content of pectin: (**a**) Fresh morphology of emulsion gel beads; The SEM pictures were taken at 30× (**b**) and (**c**) 100×.

The visual appearances, surface and tangent plane morphologies of all beads were determined. The WPI emulsion–pectin gel beads with different concentrations of pectin (20%, 40%, 50%, 60% and 80%) for the quercetin delivery system were prepared by an ionic gelation method. The particle sizes of freshly prepared beads depended on the pectin content, and their diameter was significantly increased when the pectin content increased from 20% to 80% (Table 1). Researchers have reported that the higher the viscosity of the polymer solution, the slower the flow through the nozzle of the injector and the larger beads were prepared (Lee, et al., 2020). However, gel beads diameters during the freeze drying became smaller than those of freshly prepared beads due to the water loss. Meanwhile, Figure 5 described the shape and appearance of the beads obtained, it could be seen that the formability of the pellets gets better and better as the content of pectin increased. Moreover, it should be pointed that yellow of beads was the color of quercetin (Figure 5a), indicating that quercetin was successfully encapsulated. It could be clearly seen that color of gel beads became more transparent with the content of pectin increased.

The differences in the surface morphology and internal structure of quercetin containing beads (after they were lyophilized) from observing SEM micrographs exhibited in Figure 5b. All hydrogel beads were spherical or slightly oval-shaped, especially beads with high content of pectin were irregular shape because the shape of beads was wrinkled and exhibited collapse to some extent during freeze-drying. 20% pectin beads had the most regular spherical shape and porous surface, without obvious collapsed structure. The reason might be that WPI emulsion was dominate in the system and oil droplets hindered the water accessed to structure. The previous study had demonstrated that increasing the content of protein could reduce water loss and shrinkage [35]. Additionally, it could be seen that there were some large holes from tangent plane of beads (Figure 5c), suggesting that quercetin could be easily to release than other beads. By increasing the content of pectin mixed with WPI emulsion, leading the internal and external structures of the beads changed due to the interstitial spaces of the systems was occupied by pectin. From the SEM, it was exhibited that the surface morphology was more and more smooth and tighten among high content pectin beads, cavities became smaller and smaller, which was consistent with the results of previous study [36].

### 2.6. Encapsulation Efficiency and In Vitro Release

**Figure 6:**
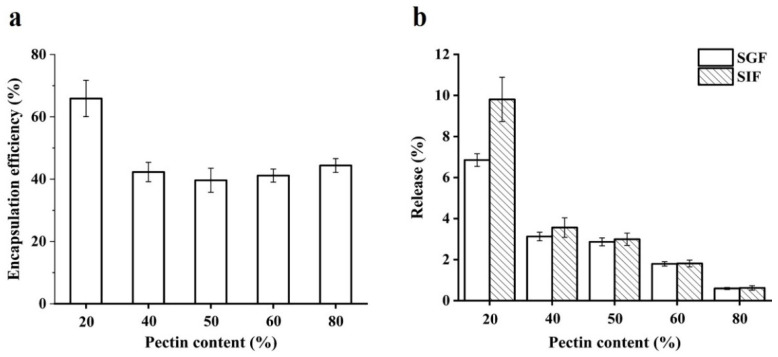
Encapsulation efficiency of quercetin from beads with different content of pectin (**a**); the release amount of quercetin from beads with different content of pectin in simulated gastrointestinal fluids (**b**). SGF and SIF represent simulated gastric fluid and simulated intestinal fluid, respectively.

The content of quercetin in WPI emulsion–pectin beads was measured. Compared with WPI emulsion, the encapsulation efficiency of all beads was improved (Figure 6a). The encapsulation efficiency of quercetin in beads exhibited high values at 20% pectin. However, the encapsulation efficiency decreased when pectin concentration from 20 to 80% and there was no difference among them. The cross-linking between WPI emulsion and pectin could provide a more compact structure than WPI emulsion, resulting in a diffusion barrier of quercetin. Therefore, in the present study, a formation of complex polymer through cross-linking led to excellent entrapment of quercetin in hydrogel beads.

The in-vitro release study was aimed to reveal that quercetin could be released slowly from the beads. It was found that the release of quercetin from all samples was below 10% after exposure to simulated gastric fluid (SGF) and simulated intestinal fluid (SIF) in the Figure 6b. These results indicated that the beads could protect the release of quercetin. It had been reported that the hydrolysis of pectin was mainly disintegrated by intestinal microorganism [24], which made quercetin release hard from the hydrogel beads. The research had reported that the de-esterified yuzu peel pectin was used to prepare hydrogel beads as quercetin delivery systems, the quercetin release after exposure to SGF and SIF was below 1% but it was greatly released (65.37 and 99.54%) when it was exposed to simulated colonic fluid [27]. In conclusion, WPI emulsion–pectin gel beads might be an effective delivery system for quercetin to colon target, which should be worth to further study.

## 3. Conclusions

In this study, a core-shell bioactive delivery system using natural food grade materials was successfully constructed. Beads were fabricated by mixing WPI emulsion with pectin under the ionotropic gelation method. FTIR, TGA and texture methods were used to investigate the effect of pectin content on the properties of beads the gel beads, confirming that incorporation of high pectin content into gel beads affect their texture, mesh-like structure and thermodynamics. These findings are important for producing emulsion gel beads during gelation because the properties of emulsion gel beads may affect encapsulation, stability, and release of hydrophobic functional ingredients embed in emulsion gel beads. As for in vitro digestion, it was found that beads loading quercetin were stable in SGF and SIF, indicating indirectly that it might be a promising delivery system to colon target for quercetin release. Nonetheless, the delivery systems for colon target should be further explored deeply. To sum up, these results might provide valuable information for nutraceutical delivery systems in functional food industry.

## 4. Materials and Methods

### 4.1. Materials

Whey protein isolate (WPI) was purchased from American Davisco chemical co., LTD. The product contained 97.6% protein (dry basis), as determined by the supplier’s standard proximate analysis procedures. Medium-chain triglyceride (MCT) oil and low-methoxy pectin were bought from Yuanye Bio-Technology Co., Ltd. (Shanghai, China) and Solarbio Co. Ltd. (Beijing, China), respectively. Quercetin, pepsin, lipase, pancrelipase and bile salts were purchased from Sigma-Aldrich (Shanghai, China). Sodium chloride (NaCl), hydrochloric acid (HCl), sodium hydroxide (NaOH) and calcium chloride (CaCl_2_) were bought from Sinopharm Chemical Reagent Co., Ltd. (Beijing, China). All chemicals were of analytical grade and the water used in this study was deionized.

### 4.2. Preparation of Emulsions and Emulsion Gel Beads Containing Quercetin

The dispersions of WPI (10 wt%) and pectin (2 wt%) were stirred at room temperature for 2 h using a magnetic stirrer, respectively, and then pH values of dispersions were adjusted to 7.0 with 1 M HCl and NaOH. These solutions were kept overnight to ensure complete dispersion and dissolution.

For production of quercetin emulsion: WPI stabilized quercetin emulsion (10 wt% protein) was prepared with 10 wt% medium chain triacylglycerol (MCT) oil containing quercetin (quercetin was firstly dissolved in MCT oil at 45 °C for 30 min, 0.02 wt% in the final emulsion) as the dispersed phase and 90 wt% aqueous phase solution at room temperature. The quercetin emulsion was prepared using an Ultra-Turrax (IKA-25, Staufen, Germany) at a speed of 10,000 rpm for 3 min to form coarse emulsions, which were subsequently homogenized by using an ultrasonic cell disruptor (Biosafer3D, Saifei Technology Co., Ltd., Hangzhou, China) at ultrasonic power was 300 W for 10 min.

For production of emulsion gel beads: the above emulsions mixed with pectin solution (finally the content of pectin was 20%, 40%, 50%, 60% and 80% in the mixture) at room temperature for 1 h, and then they were dropped into 2% (*w*/*w*) calcium chloride solutions by using 5 mL measuring pipets and the distance between the tip of pipet and the surface of CaCl_2_ solutions was fixed at 10 cm, respectively. The samples were allowed to gel in CaCl_2_ solutions for 30 min with mild magnetic stirring, and then beads were rinsed with distilled water.

### 4.3. Physical Properties of Bead Forming Solution

#### 4.3.1. Surface Zeta-Potential

The ζ-potential of all solutions for preparing beads were measured by using Zetasizer Nano-ZS90 (Malvern Instruments, Worcestershire, UK). All stock solutions were diluted to 500 folds using distilled water (the resistivity was 15 MΩ cm) to avoid multiple scattering effects. After loading the samples into the instrument, they were equilibrated for about 120 s before particle charge data was collected over 11 continuous readings.

#### 4.3.2. Rheology

The apparent viscosity of stock solutions was measured by using a dynamic shear rheometer (Rheometer MCR 301 Anton Paar, Graz, Austria) with a parallel plate geometry (CP 50-1: diameter of 50 mm, and the gap of 1 mm) at 25 °C. The samples were placed on the plates and equilibrated for 2 min before testing. The apparent viscosity of sample was calculated when shear rates were ranging from 2 to 200 s^−1^.

### 4.4. Textural Properties and Water Content

The texture profile analysis (TPA) tests were performed by using a texture analyzer (model TA-XT2i, Stable Micro Systems Ltd., Surrey, UK) equipped with a cylindric shaped probe (P36R probe, a diameter of 25 mm). The freshly prepared spherical beads (about 2 g) were placed on a fixed bottom plate under the probe at 50% compression. The pre-test, test and post-test speeds were set at 1.0 mm/s, 0.5 mm/s and 1 mm/s, respectively. Furthermore, trigger force was 1 g, and the time between two loadings was 5 s. The texture parameters including hardness, springiness, cohesiveness, resilience, gumminess and chewiness values of beads are calculated by the software Texture Expert Version 1.22.

The water content of beads with different pectin content was detected with Moisture Analyzer (HE 53, Mettler Toledo, Guangzhou, China). The fresh gel beads (about 0.8 g) were placed on the plate made by aluminized paper for measuring.

### 4.5. Fourier Transform Infrared Spectroscopy

Fourier transform infrared spectroscopy (FT-IR) spectra was used for detecting WPI, quercetin, pectin and freeze-dried beads. All freeze-dried samples were examined on an FT-IR spectrometer (IRTracer-100, Shimadzu Corp., Kyoto, Japan). Firstly, all samples should be mixed with KBr reagent (a mass ratio of 1:50) and were ground into powders. Next, the powders were compressed into disks by using Hydraulic press (GS03940, Specac, Washington, DC, USA) at 1.7 tons, which are used for FTIR detection. The wavenumber of FT-IR spectrum was between 4000 and 400 cm^−1^ and 60 scans at a resolution of 4 cm^−1^, and all spectra of the prepared disks were recorded.

### 4.6. Thermo-Gravimetric Analysis (TGA) and Swelling Study

The thermal stability of freeze-dried beads was measured by using Thermogravimetry Analyzer (SDT Model Q600, TA Instrument, New Castle, DE, USA). The sample was placed on a platinum pan and the temperature was raised from 30 °C to 500 °C and the heating temperature was scanned at the rate of 10 °C/min. The weight loss was monitored with respect to time and temperature.

The swelling behavior of the freshly prepared beads was detected according to the methods previously described [13,37]. Briefly, 0.5 g of beads was immersed into distilled water and incubated at room temperature for different time (0.5 h, 1 h, 1.5 h, 2 h, 3 h, 5 h and 6 h) and then the beads were withdrawn from water and remove excess surface fluid with filter paper. The mass of the swollen beads was recorded. The percentage swelling ratio was calculated by following equation (Equation (1)):

Swelling ratio (%) = [(W_s_ − W_i_)/W_i_] × 100 (1)

where W_s_ and W_i_ are the weight of the swollen beads and initial beads, respectively.

### 4.7. Scanning Electron Microscopy Analysis

The morphologies of freeze-dried gel beads were analyzed by a scanning electron microscope (SEM, S-4800, Hitachi, Tokyo, Japan) using an accelerating voltage of 5 kV under low vacuum conditions. The surface and internal (cut with a blade) morphologies of freeze-dried gel beads were examined. The beads were affixed to conductive carbon tapes and coated with a thin layer of gold by a sputter coater before observation. Moreover, gel beads diameters were measured using Leica Application Suite EZ software 3.4.0.

### 4.8. Quecetin Encapsulated Efficiency and In-Vitro Release Study

The amount of quercetin present in beads was quantified using an Ultraviolet spectrophotometer (UV mini-1240 Mettler Toledo Instruments Co., Ltd., Shanghai, China). Before measuring, all beads were added into 2% sodium citrate solution until they were dissolved completely. Subsequently, referring previous study to measure the amount of quercetin [8]. Briefly, one milliliter of sample from the middle of the bottle was dissolved in DMSO (ratio 1:10) then centrifuged at 3000 rpm for 15 min because DMSO could increase the solubility of quercetin in the aqueous phase. Next, the supernatant was filtered using a syringe filter with a mean pore size of 0.45 μm (Toyo Roshi Kaisha Ltd., Tokyo, Japan). The quercetin concentration was then determined by measuring the absorbance at 372 nm. The encapsulation efficiency (EE) of quercetin encapsulated in beads was calculated using the following equation (Equation (2)):

EE = (C_d_/C_i_) × 100 (2)

where C_d_ is the quercetin concentration that remained in the sodium citrate solution and C_i_ is the initial quercetin concentration.

The quercetin-loaded gel beads were used to explore the release behavior in vitro digestion. The method was according to previous research [38]. Briefly, the beads (1 g) were dispersed in 20 mL simulated gastric fluid (SGF, 3.2 mg/mL of pepsin, 0.2% of NaCl, 0.7% of HCl, pH 2.0) and incubated for 2 h at 37 °C with shaking. Then, the pH of the mixture was adjusted to 6.8 and then 20 mL of simulated intestinal juice (SIF, 0.4 mg/mL of lipase, 0.5 mg/mL of pancreatin, 0.7 mg/mL of bile extract, pH 6.8) was mixed with the above mentioned SGF. This mixture incubated at 37 °C and shook at 100 rpm for 4 h. The amounts of quercetin released from beads were determined with the above method.

## Figures and Tables

**Table 1:** Mean particle diameters (mm) for emulsion beads produced by different contents of pectin before and after freeze drying. Different letters (a to d) indicate significant differences (*p* ≤ 0.05).

Pectin Content	Mean Particle Diameter (mm)	
	Fresh	Freeze Drying
20%	3.84 ± 0.07 ^a^	3.57 ± 0.11 ^a^
40%	3.98 ± 0.03 ^b^	3.81 ± 0.05 ^b^
50%	4.04 ± 0.02 ^b,c^	3.97 ± 0.05 ^c^
60%	4.08 ± 0.02 ^c^	3.96 ± 0.06 ^c^
80%	4.16 ± 0.04 ^d^	3.97 ± 0.04 ^c^

## Data Availability

The data presented in this study are available on request from the corresponding author.
